# A Hybrid Quantum-Classical Model for Stock Price Prediction Using Quantum-Enhanced Long Short-Term Memory

**DOI:** 10.3390/e26110954

**Published:** 2024-11-06

**Authors:** Kimleang Kea, Dongmin Kim, Chansreynich Huot, Tae-Kyung Kim, Youngsun Han

**Affiliations:** 1 Department of AI Convergence, Pukyong National University, Nam-gu, Busan 48513, Republic of Korea; kimleangkea@pukyong.ac.kr (K.K.); kdm902077@pukyong.ac.kr (D.K.); huotnich@pukyong.ac.kr (C.H.); 2 Department of Management Information Systems, Chungbuk National University, Seowon-Gu, Cheongju 28644, Republic of Korea

**Keywords:** AI in finance, long short-term memory, quantum machine learning, stock price prediction, time-series analysis

## Abstract

The stock markets have become a popular topic within machine learning (ML) communities, with one particular application being stock price prediction. However, accurately predicting the stock market is a challenging task due to the various factors within financial markets. With the introduction of ML, prediction techniques have become more efficient but computationally demanding for classical computers. Given the rise of quantum computing (QC), which holds great promise for being exponentially faster than current classical computers, it is natural to explore ML within the QC domain. In this study, we leverage a hybrid quantum-classical ML approach to predict a company’s stock price. We integrate classical long short-term memory (LSTM) with QC, resulting in a new variant called QLSTM. We initially validate the proposed QLSTM model by leveraging an IBM quantum simulator running on a classical computer, after which we conduct predictions using an IBM real quantum computer. Thereafter, we evaluate the performance of our model using the root mean square error (RMSE) and prediction accuracy. Additionally, we perform a comparative analysis, evaluating the prediction performance of the QLSTM model against several other classical models. Further, we explore the impacts of hyperparameters on the QLSTM model to determine the best configuration. Our experimental results demonstrate that while the classical LSTM model achieved an RMSE of 0.0693 and a prediction accuracy of 0.8815, the QLSTM model exhibited superior performance, achieving values of 0.0602 and 0.9736, respectively. Furthermore, the QLSTM outperformed other classical models in both metrics.

## 1. Introduction

Predicting involves forecasting future events by analyzing historical data. Forecasting stock prices is a challenging task, as they are influenced by factors such as political conditions, the global economy, and company performance [1]. Research into stock price prediction predominantly relies on two types of data: time-series structured historical market data and unstructured textual sources, like financial news [2]. A time-series, in this context, is a chronological sequence of observations for a specific variable. In our scenario, the stock price is a time-series dataset as it involves daily historical records, including opening, closing, highest, and lowest prices, alongside trading volumes. Traditionally, the primary method for forecasting a company’s stock price has been through technical analysis. This method utilizes historical data such as closing and opening prices, trading volume, and adjacent close values to predict future stock price data [3].

Recently, machine learning (ML), particularly deep learning (DL), has achieved tremendous success in various fields, particularly computer vision [4,5] and natural language processing [6]. Notably, White et al. [7] was the first to successfully predict stock market time-series using the backpropagation neural network (BP-NN). Subsequently, Kolarik et al. [8] compared the prediction results of artificial neural networks (ANN) with those of the auto-regressive integrated moving average model (ARIMA), demonstrating the superior effectiveness of ANN. In stock price prediction, two commonly used DL architectures are recurrent neural networks (RNNs) and long short-term memory (LSTM) networks. LSTM, characterized by a sequence of memory cells operating as computational units, is particularly adept at analyzing and forecasting time-series data with remarkable accuracy and efficiency. Chen et al. [9] achieved stock returns prediction with the LSTM model, while Fischer et al. [10] utilized LSTM to predict stock prices and devise short-term investment strategies. However, these architectures are limited by several factors, such as vanishing and exploding gradients, as well as high computational complexity and intensity.

In recent years, quantum computing (QC) technology has attracted considerable attention, encompassing both hardware and quantum algorithm development [11,12]. Theoretically and evidently, quantum computers have the potential to solve problems that are currently beyond the capabilities of classical computers, such as factoring large integers and implementing quantum search algorithms [13]. However, quantum computers are still in their infancy, hindered by the low availability of quantum bits (qubits) and high physical device error rates. Therefore, the utilization of hybrid quantum-classical algorithms, such as the variational quantum eigensolver, quantum approximate optimization algorithm, and quantum machine learning (QML), has gained traction. These hybrid algorithms employ variational quantum circuits (VQCs), a type of quantum circuit whose gate parameters are adjustable and optimized classically, effectively harnessing the computational resources of both quantum and classical computers [14]. However, the major challenge for VQCs is related to “barren plateaus,” which are large regions where the cost function is flat and therefore untrainable by any gradient-based learning algorithm [15].

To harness the advantages of classical LSTM in analyzing stock price time-series data and leverage the expressive capabilities of QC, a hybrid quantum-classical computing model is developed for predicting stock prices. This hybrid framework combines a classical LSTM model for thorough data analysis with a VQC for quantum-enhanced execution, aiming to predict stock prices with excellent accuracy and efficiency. The main contributions of this study are summarized below:

- We introduced a specifically designed hybrid quantum-classical computing framework for predicting the stock price data of a single company.
- We detailed and utilized the quantum circuit for encoding classical data into quantum states, potentially leading to more efficient representations that better capture the underlying patterns in stock price data.
- We also offered a thorough discussion on limitations and proposed future works to extend its applicability beyond stock price prediction, encompassing other domains, particularly focusing on time-series data analysis.
- Our experiments illustrate the superior performance of the proposed QLSTM model over classical LSTM and other specialized time series models. It achieves a significantly decreased root mean square error (RMSE) and an improved accuracy, evident throughout both the training and prediction phases.
- We rigorously evaluated the QLSTM model by executing it on an IBM quantum simulator and further validated its capabilities by conducting predictions on actual IBM quantum hardware. Additionally, we conducted tests under various quantum environments, including noiseless and noisy quantum simulators, showcasing its remarkable performance superiority.

The remaining sections are organized as follows. Section 2 briefly presents the background of classical ML and QC. Section 3 describes the related works of both classical and quantum models for time-series data prediction. Then, the utilized QLSTM model architecture is introduced in Section 4. Section 5 shows the experimental results of the proposed method with the stock price data. Section 6 provides a discussion and limitations of our studies. The conclusion is provided in the last Section 7.

## 2. Background

In this section, we introduce the fundamental concepts of classical ML and QC, setting the stage for a discussion on its quantum counterparts.

### 2.1. Classical Machine Learning

**Figure 1:**
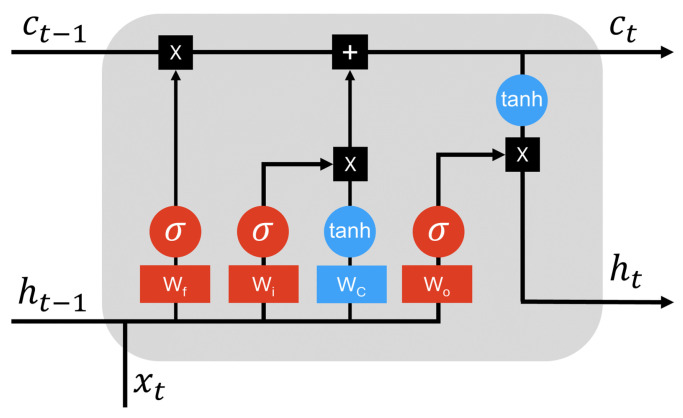
Schematic for the internal structure of a classical LSTM cell.

RNNs are a class of models in classical ML capable of handling sequential data by memorizing the history of previous data inputs to make more accurate predictions [16]. Unlike traditional feedforward networks, an RNN produces an output for the current time step and maintains a hidden state that cycles back into the network, constructively retaining information from previous time steps. However, as the RNN depth or the sequence length increases, it often faces the challenge of vanishing or exploding gradients [17]. LSTM networks are an improved version of RNNs, solving the problems encountered by RNNs through small modifications to the information through multiplications and additions. With LSTM, data propagation flows through cell units, facilitating retention or the discarding of information selection. It consists of a series of interconnected cells arranged in an unrolled manner, enabling the processing of sequential data effectively [18]. Each cell, at time step *t*, receives three distinct inputs: $x_{t}$, $h_{t-1}$, and $c_{t-1}$, and consequently generates two outputs: $h_{t}$ and $c_{t}$. As shown in Figure 1, the architecture of an LSTM cell, which relies on three gates to control the cell state, is mathematically represented as follows:

(1) $$\begin{array}{cc} f_{t} & =\sigma (W_{f}\left[h_{t-1},x_{t}\right]+b_{f}),\\ i_{t} & =\sigma (W_{i}\left[h_{t-1},x_{t}\right]+b_{i}),\\ {\tilde{C}}_{t} & =tanh(W_{C}\left[h_{t-1},x_{t}\right]+b_{C}),\\ c_{t} & =f_{t}*c_{t-1}+i_{t}*{\tilde{C}}_{t},\\ o_{t} & =\sigma (W_{o}\left[h_{t-1},x_{t}\right]+b_{o}),\\ h_{t} & =o_{t}*tanh\left(c_{t}\right), \end{array}$$

where σ denotes the sigmoid function, $W_{f}$, $W_{i}$, $W_{o}$, and $W_{C}$ are classical NNs for forget gate, input gate, output gate, and cell state, respectively, $b_{f,i,o,C}$ is the corresponding biases for the $W_{f,i,o,C}$, while $\left[h_{t-1},x_{t}\right]$ refers to the concatenation of hidden state $h_{t}$ and $x_{t}$ to each cell, respectively. The input gate $i_{t}$ determines the new information to add to the cell state $c_{t}$, while the cell state $c_{t}$ retains relevant information over time. The output gate $o_{t}$ controls the output $h_{t}$ based on the cell state $c_{t}$. Additionally, ${\tilde{C}}_{t}$ is the candidate cell state, which is created to enhance the cell state update.

### 2.2. Quantum Computing

In classical computing, a binary unit of information is stored in a bit, which can take one of two values: 0 or 1. Bit operations are performed using elementary logic gates, such as AND, OR, and NOT. Conversely, QC benefits from quantum bits (qubits) which can hold combinations of 0 and 1 at the same time via superposition and entanglement. Simultaneously, the two qubit states |0〉 and |1〉 can be defined as follows:

(2) $$|0\rangle =\left[\begin{array}{c} 1\\ 0 \end{array}\right]\;|1\rangle =\left[\begin{array}{c} 0\\ 1 \end{array}\right]$$

Any qubit |ψ〉 can be described as a linear combination of two basis states, α|0〉+β|1〉, where α and β are complex numbers, and ${\left|\alpha \right|}^{2}+{\left|\beta \right|}^{2}=1$. As a qubit can exist as a superposition of two classical states, two qubits allow the superposition of four states, and *n* qubits allow the superposition of $2^{n}$, represented by $\left|0\rangle ,|1\rangle ,|2\rangle ,\ldots \;\right|2^{n}-1\rangle$. In a multi-qubit system, where all qubits are in superposition, the state of one qubit can become correlated with the state of another. As a result, changing or measuring one qubit reveals the value of the other. This phenomenon is known as entanglement [19]. Additionally, the total probability across all qubits in superposition equals 1. At the same time, two strategies to achieve quantum advantage are mentioned in a study [13]. The first involves showing that current quantum devices can execute computations that are beyond the reach of classical simulations. The second strategy focuses on developing quantum circuits tailored to specific problems, leveraging these devices for a computational advantage In this paper, we prioritize the first approach, emphasizing the efficiency of quantum computers in handling large-scale high-dimensional data in polynomial time [20,21], making them particularly promising for machine learning applications [22].

### 2.3. Quantum Machine Learning

**Figure 2:**
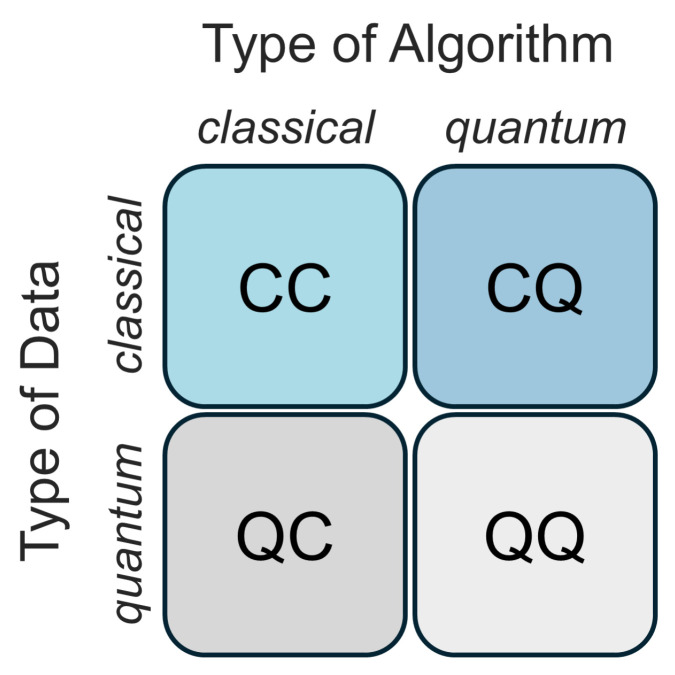
Four different methods for integrating ML with QC.

ML is among the most successful and extensively researched technologies in computer science [23]. Consequently, it has been integrated with QC to form QML, which aims to solve complex classical ML problems by harnessing the unique computational capabilities of QC [24]. There are four strategies for integrating ML and QC, based on whether one considers the data to have been created by a classical (C) or quantum (Q) system, and whether the computer processing the data is classical (C) or quantum (Q), as shown in Figure 2.

The first scenario, CC, involves utilizing classical data with traditional ML algorithms. In contrast, the second scenario, QC, explores the potential of leveraging ML to enhance quantum computers by analyzing quantum measurement data. The CQ scenario uses quantum algorithms to examine classical datasets. This approach requires translating classical data into quantum data, a process known as quantum encoding. The last scenario, QQ, involves the processing of quantum data by a quantum device using a quantum algorithm. In our study, we concentrate on the CQ scenario. This approach involves running QML algorithms on quantum simulators or computers to achieve algorithmic superiority [24]. Moreover, there are many approaches exist which can be employed to maximize the potential of hybrid CQ methods. These typically begin with encoding classical data into quantum states which refers to data encoding. Followed by data encoding, a variational quantum circuit with fixed parameters is constructed, which enables the approximation, optimization, and classification of various computational tasks [25].

### 2.4. Quantum Encoding

**Figure 3:**
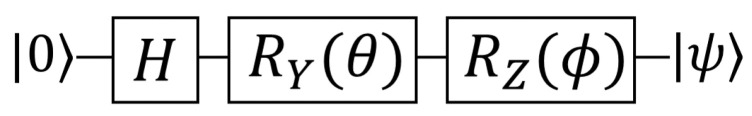
Angle encoding quantum circuit.

Several QML algorithms require converting classical data into quantum states within quantum computers, a process known as data encoding [26]. In other words, the dataset is initially translated from the subject data domain *D* to the Hilbert space *H* through a designated feature mapping process f:D→H. One of the encoding techniques, specifically angle encoding or qubit encoding, is widely renowned for its ability to leverage a minimal number of qubits that correspond to the size of the input vector [27]. The angle encoding scheme demonstrates remarkable efficiency as it necessitates the rotation of only a single qubit as depicted in Figure 3.

In this technique, each data value *x* undergoes an initial normalization process, mapping it to the range [0,2π]. Subsequently, it is encoded using single-qubit rotation gates $R_{X}$, $R_{Y}$, or $R_{Z}$. These rotation gates dynamically determine the rotation angle θ based on the corresponding data value *x*. As such, this approach requires *n* qubits to encode *n* input variables defined as follows:

(3) $$|\psi_{x}\rangle =\overset{n}{\underset{i=1}{⨂}}R\left(x_{i}\right)|\psi_{0}\rangle ,$$

where *R* is one of the rotation gates and $\psi_{0}$ is an initial state. The tensor product ⨂ signifies that the quantum state is a multi-qubit state formed by the individual states created by each rotation gate.

## 3. Related Works

In this section, we will review several relevant research works that explore financial applications, especially stock price prediction, using both classical ML and QML.

### 3.1. Classical Machine Learning

Chen et al. [28] proposed a two-stage portfolio-selection method using ML for stock price prediction. First, they adopted classical ML models to forecast stock prices across various datasets. Thereafter, they applied mean-variance portfolio optimization to identify the most promising stocks for investment based on higher potential returns within the predictions. The experimental results yielded RMSE values of 0.0744 and 0.0807 for LSTM and ANN, respectively, highlighting the superior performance of LSTM in error minimization. However, the prediction results are not as robust as desired and could, consequently, influence portfolio selection. Mehtab et al. [29] designed and evaluated eight ML-based regression models for stock price prediction, including multivariate linear regression, multivariate adaptive regression spline, regression tree, bootstrap aggregation, extreme gradient boosting, random forest, ANN, and support vector machine (SVM). Notably, the LSTM-based DL models outperformed other ML regression models significantly. However, their designed LSTM model performed poorly with multivariate data.

Khan et al. [30] applied algorithms to analyze the impact of social media and financial news data on stock price prediction accuracy over 10 days. They conducted experiments to identify unpredictable stock markets, comparing various algorithms to find a consistent classifier. Thereafter, DL was applied to obtain the maximum accuracy, with some classifiers combined through ensembling. The experimental results demonstrate that the most accurate predictions, reaching 80.53% and 75.16%, respectively, are attained through the analysis of social media and financial news. Hamayel et al. [31] proposed three types of RNN algorithms for predicting the prices of three types of cryptocurrencies, namely gated recurrent unit (GRU), LSTM, and bi-LSTM. Based on the outcomes, the GRU model for the targeted cryptocurrencies was considered efficient and reliable. The experiment yielded RMSE values of 3.069, 4.307, and 0.825 for LSTM, bi-LSTM, and GRU, respectively. However, GRUs may not be as effective at storing and accessing long-term dependencies as LSTMs due to their simpler structure and fewer gating mechanisms.

### 3.2. Quantum Machine Learning

Here, we present a curated selection of works focusing on the application of QML in analyzing and predicting time-series data, particularly on stock price data. Emmanoulopoulos et al. [32] investigated the performance of an approach utilizing parameterized quantum circuits as quantum neural networks (QNNs) for forecasting time-series data. The performance of the QNNs was compared to that of a classical bi-LSTM model to evaluate their effectiveness. However, we did not consider using quantum noise and an actual quantum machine to validate the model’s effectiveness. While several studies have proposed reservoir quantum computing as an alternative technique for time-series prediction [33,34,35], this work focuses exclusively on the QNN approach. We aim to explore the advantages of QNNs without delving into reservoir quantum computing. Srivastava et al. [36] investigated quantum algorithms for stock-price prediction through experimental simulations on both classical and actual quantum machines. They employed quantum annealing for feature selection and principal component analysis for the dimensionality reduction of the data. The prediction task was transformed into a classification problem, and a quantum SVM was trained to predict the stock prices. However, the experimental accuracy was only 60%; this is expected since quantum SVMs are not particularly suitable for prediction tasks. Paquet et al. [37] introduced a hybrid QNN called QuantumLeap for financial prediction. The network comprised an encoder that transforms partitioned financial time series into a sequence of density matrices, a deep quantum network that predicts the density matrix at a later time, and a classical network that measures the maximum price reached by the security at that time based on the output density matrix. The experimental results demonstrate the prediction accuracy and efficiency of the model. However, the proposed design is computationally intensive, potentially posing challenges in terms of scalability and practical implementation.

## 4. Stock Price Prediction Using QLSTM

Here, we detail the development of the QLSTM architecture for predicting stock price by seamlessly integrating a VQC with a modified classical LSTM model.

### 4.1. Overall Architecture

**Figure 4:**
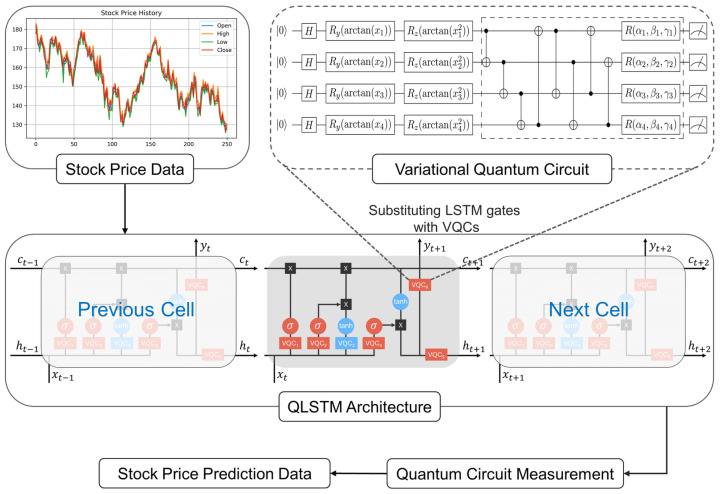
The overall architecture of the proposed QLSTM for stock closing price prediction.

In this study, we developed a QLSTM model following the procedures introduced in [38], which is potentially applicable to noisy intermediate-scale quantum (NISQ) devices. The process comprised the following stages: (i) initialization of input data, (ii) encoding input data into a quantum state, (iii) employing quantum gates to manipulate the quantum state, (iv) measuring the quantum gates, and (v) generating output predicated on the measured results [39]. The overall framework is illustrated in Figure 4, wherein stock price data are collected and inputted into the QLSTM for training. Thereafter, the collected data undergo common preprocessing operations, such as normalization and transformation. As it is a hybrid quantum-classical model, classical NNs and VQCs are initialized by specifying the number of features, layers, and qubits, after which the model is prepared for the quantum encoding of the stock-price dataset. Subsequently, the VQCs perform the required computations by rotating and entangling the qubits. The computation results of the VQCs are measured and postprocessed to obtain the predicted price. This process iterates multiple times to minimize prediction errors, and the loss between the predicted and actual prices is calculated. By combining classical and quantum computing elements, this hybrid approach aims to leverage the power of quantum-enhanced learning for more accurate and efficient stock price prediction.

### 4.2. QLSTM Structure

**Figure 5:**
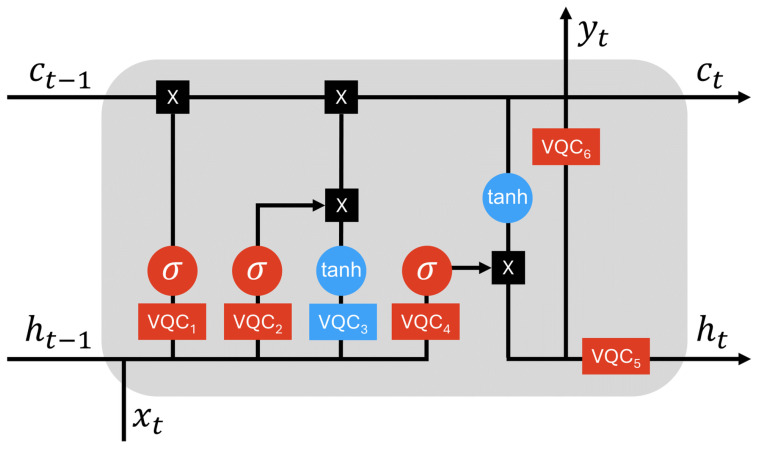
A QLSTM cell consists of VQCs as replacements to LSTM gates.

The main difference between the quantum and LSTM architecture is the type of network architecture: QLSTM utilizes VQCs for computation, whereas LSTM relies on linear recurrent neural networks. Although the QLSTM model is theoretically more efficient than the LSTM model, it remains in the early stages of development. In designing the QLSTM model, we replicate the behavior of the LSTM cell using quantum gates. As illustrated in Figure 5, each VQC box resembles an LSTM cell gate. Specifically, *VQC1* to *VQC4* correspond to the forget, input, update, and output gates, respectively. *VQC5* is used to convert the cell state $c_{t}$ to the hidden state $h_{t}$, whereas *VQC6* is utilized to further refine the cell state $c_{t}$ into the output $y_{t}$. Finally, the output $y_{t}$ is derived from the measurements taken at the end of each VQC. These measurements yield Pauli *Z* expectation values for each qubit involved. Optionally, these values can undergo nonlinear activation functions during classical post-processing, thereby reshaping the final output. The structure is depicted in Figure 5, with the corresponding mathematical formulations presented as follows:

(4) $$\begin{array}{cc} v_{t} & =[h_{t-1},x_{t}],\\ f_{t} & =\sigma (VQC_{1}\left(v_{t}\right)),\\ i_{t} & =\sigma (VQC_{2}\left(v_{t}\right)),\\ {\tilde{C}}_{t} & =tanh(VQC_{3}\left(v_{t}\right)),\\ c_{t} & =f_{t}*c_{t-1}+i_{t}*{\tilde{C}}_{t},\\ o_{t} & =\sigma (VQC_{4}\left(v_{t}\right)),\\ h_{t} & =VQC_{5}\left(o_{t}*tanh\left(c_{t}\right)\right),\\ y_{t} & =VQC_{6}\left(h_{t}\right), \end{array}$$

where $v_{t}$ is defined as the concatenation if the previous hidden state $h_{t-1}$ and the current input $x_{t}$ and $y_{t}$ denotes the newly introduced output incorporated into QLSTM model.

All VQCs have been formulated and categorized into three blocks within the QLSTM architecture. These blocks function analogously to gates within the classical LSTM model. Detailed information is provided below:

- **Forget Gate**: The $VQC_{1}$ box processes the concatenated hidden state $h_{t}$ alongside input data $x_{t}$ to produce a vector $f_{t}$ using the sigmoid function. This vector contains values between 0 and 1. The role of $f_{t}$ is to dictate whether to preserve or eliminate corresponding elements in the cell state $c_{t-1}$ from the previous time step. This is executed through element-wise operations applied to $c_{t-1}$. Assigning a value of 1 indicates full retention of the corresponding element within the cell state, while a value of 0 signifies forgetting. However, in QLSTM, the operations on the cell state is not limited to 0 or 1 but encompass a linear combination between them, making QLSTM suitable for efficiently learning temporal dependencies.
- **Input and Update Gates**: The goal of these gates is to determine the new information to be added to the cell state. First, $VQC_{2}$ processes the input $v_{t}$, passing the output through the sigmoid function to determine which values will be incorporated into the cell state. Concurrently, $VQC_{3}$ processes the concatenated input and undergoes a transformation via a hyperbolic tangent (tanh) function, generating a new cell state candidate ${\tilde{C}}_{t}$. Subsequently, the output from $VQC_{2}$ is element-wise multiplied by ${\tilde{C}}_{t}$, and the resultant vector is used to update the cell state.
- **Output Gate**: First, $VQC_{4}$ processes the input $v_{t}$ by passing it through the sigmoid function to determine the relevance of values in the cell state $c_{t}$. Following this, the cell state is transformed via the hyperbolic tangent function (tanh) and then multiplied by the output of $VQC_{4}$. Optionally, the resulting value can undergo further processing through $VQC_{5}$ to generate the hidden state $h_{t}$, or through $VQC_{6}$ to yield the output $y_{t}$. In general, the dimensions of the cell state $c_{t}$, the hidden state $h_{t}$, and the output $y_{t}$ are not identical. To ensure correct dimensions, we utilize $VQC_{5}$ to transform $c_{t}$ into $h_{t}$, and $VQC_{6}$ to transform $c_{t}$ into $y_{t}$, respectively.

### 4.3. Variational Quantum Circuits

**Figure 6:**
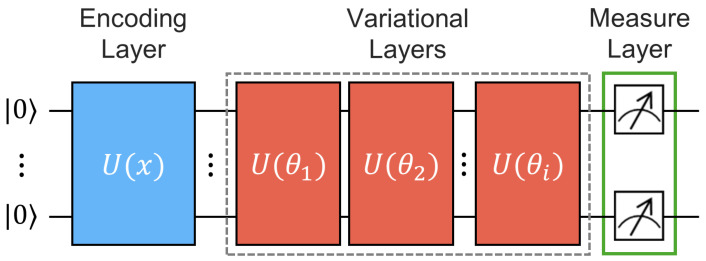
The general architecture for a single variational quantum circuit (VQC) is described as follows: U(x) represents the quantum operations for encoding classical data (*x*), and U(θ) represents the repetition of variational layers, from 1 to *i*, each with tunable parameters θ. The final layer is a measurement layer employed to obtain the VQC probability distribution.

A VQC is a quantum computation model with adjustable and tunable parameters, which undergo further iterative optimizations. Typically, VQCs are structured with three fundamental layers, as depicted in Figure 6: data encoding, variational, and measurement layers. The computational process is accomplished by encoding classical data into quantum states using a sequence of quantum gates. The U(x) block is responsible for quantum state preparation, encoding the classical data *x* into the quantum state of the circuit, and is not subject to optimization, whereas the U(θ) block represents the variational layer with learnable parameters θ that will be optimized through gradient methods. Finally, the outcome of the computation is measured at the conclusion of the circuit. In the NISQ era, this type of circuit is robust against quantum noise since the variational layer can be extended to multiple layers. It has been successfully applied to various QML applications and has evidently demonstrated more expressive power than its classical counterparts [40]. As a result, utilizing VQCs as the building blocks of QLSTM enhances learning.

#### 4.3.1. Data Encoding Layer

This layer encodes classical data into quantum states by transitioning the qubits states from the initialized state |0〉 to the desired target states using the operation U(X). Typically, each qubit encodes one classical inputs features. A general *N*-qubit quantum state can be represented as follows:

(5) $$|\psi \rangle =\sum_{\left(q_{1},q_{2},\cdots ,q_{N}\right)\in \left\{0,1\right\}}c_{q_{1},q_{2},\cdots ,q_{N}}|q_{1}\rangle ⊗|q_{2}\rangle ⊗\cdots ⊗|q_{N}\rangle ,$$

where $c_{q_{1},q_{2},\cdots ,q_{N}}$ is the complex amplitude for each computational basis state, with $q_{i}\in 0,1$, and the addition of the square of the amplitude represents the probability distribution after measurement $|q_{1}\rangle ⊗|q_{2}\rangle ⊗\cdots ⊗|q_{N}\rangle$, ensuring that the total probability equals 1:

(6) $$\sum_{\left(q_{1},\cdots ,q_{N}\right)\in \left\{0,1\right\}}{\left\|c_{q_{1},\cdots ,q_{N}}\right\|}^{2}=1.$$

The first step of encoding in U(X) commonly utilizes the Hadamard gate *H* for encoding scheme to transform the initial state |0〉⊗⋯⊗|0〉 into an unbiased state as follows:

(7) $$\begin{array}{cc} {(H|0\rangle )}^{⊗N} & =\frac{1}{\sqrt{2^{N}}}(|0\rangle +{\left|1\rangle \right)}^{⊗N}\\ \; & =\frac{1}{\sqrt{2^{N}}}(|0\rangle ⊗\cdots ⊗|0\rangle +\cdots +|1\rangle ⊗\cdots ⊗\left|1\rangle \right)\\ \; & =\frac{1}{\sqrt{2^{N}}}\sum_{i=0}^{2^{N}-1}|i\rangle , \end{array}$$

where *N* denotes the number of qubits and *i* is the corresponding bit string within the computational basis. It is important to note that the factor $\frac{1}{\sqrt{2^{N}}}$ serves as the normalization coefficient which ensures that the total probability amplitude of the quantum state remains equal to 1.

Next, to dynamically adjust angles within the *N*-dimensional input vector $\vec{v}=\left(x_{1},x_{2},\ldots ,x_{N}\right)$, we employ rotation gates $R_{y}$ and $R_{z}$. Specifically, we utilize the arctan function to determine the angles of rotation. For $R_{y}$, we set $\theta_{i,1}=\arctan\left(x_{i}\right)$, resulting in rotation along the *y*-axis. Similarly, for $R_{z}$, we set $\theta_{i,2}=\arctan\left(x_{i}^{2}\right)$, facilitating rotation along the *z*-axis, respectively. We employ the arctan function in this context, in contrast to the arcsin and arccos functions utilized in [41]. This is because the input values typically lie in a range of real numbers, rather than within the bounded interval of [−1,1] suitable for arcsin and arccos. Additionally, squaring *x* to obtain $x^{2}$ serves to generate higher-order terms after entanglement operations.

#### 4.3.2. Variational Layer

**Figure 7:**
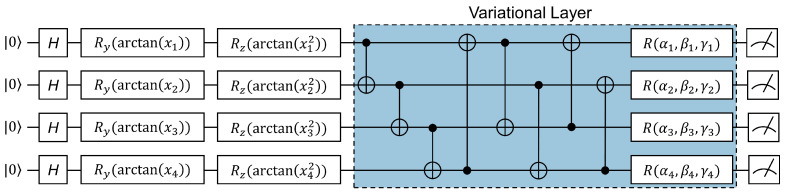
Variation quantum circuit in the QLSTM architecture, as utilized in [12,38]. *H*, $R_{y}$, and $R_{z}$ denote quantum gates, while *x* represents the classical input data vector, functioning as a data encoding layer. Parameters $\left(\alpha_{i},\beta_{i},\gamma_{i}\right)$ are adjustable and require optimization. The line connecting • and ⊗ represents a CNOT gate. The circuits conclude with a measurement layer.

In this layer, qubits are entangled and rotated to the target state using the operation U(θ), which enables nonlinear complex information mapping. Thus, the mapping properties of the variational layer can significantly influence the predictive accuracy of a variational quantum model. Since the variational layer constitutes the learnable component of a VQC, with quantum gates equipped with adjustable parameters, it can be replicated across multiple layers to further enhance the prediction performance. However, this may come at the expense of computational speed. The variational layer of the QLSTM model consists of several CNOT gates and single qubit rotation gates. However, due to the incomplete connectivity of CNOT gates across all qubit pairs, the entanglement between qubits remains insufficiently robust. To optimize the performance of the proposed model, CNOT gates are applied to every pair of qubits with fixed adjacency 1 and 2 (cyclically) to generate multiqubit entanglement. The three rotation angles, $\alpha_{i},\beta_{i},\gamma_{i}$ along the *x*, *y*, and *z* axes, respectively, in the single-qubit rotation gates $R_{i}=R\left(\alpha_{i},\beta_{i},\gamma_{i}\right)$ are not predetermined. Instead, they are adjusted during the iterative optimization process using classical gradient descent or other methods. Figure 7 illustrates the integration of VQC within the QLSTM model. The variational layer, distinguished by the cyan-colored border-box, can be iterated over multiple times to optimize performance.

#### 4.3.3. Measurement Layer

This layer generates the computational output of the VQC by measuring the quantum states of the qubits. Quantum measurements transform each qubit state into classical data, represented as 0s and 1s. In this context, we evaluate the qubit expectation values by measuring them on a computational basis. The outcome is a fixed-length vector employed for subsequent classical postprocessing. In the developed QLSTM model, these measured values from each VQC are handled within a QLSTM cell. The measured results are further processed to derive the loss function, which is employed to optimize the parameters and ultimately generate the predicted stock price data.

### 4.4. Parameter Learning

Similar to classical NNs, the parameters of VQCs can be optimized by a gradient-based approach [12,42]. However, in the context of QC, direct parameter optimization within a quantum circuit is not feasible. Consequently, the optimization of VQCs is performed with gradient computation using parameter-shift rules. Parameter-shift rules state that we can calculate the gradient of each parameter in certain quantum circuits by simply shifting the parameter twice and calculating the difference between the two outputs, all without altering the structure of the quantum circuits [43,44,45]. Given an output f(x,θ) with respect to parameter θ and input features *x*, the gradient of the VQCs can be calculated by the parameter-shift rules, as follows:

(8) $$\nabla_{\theta }f\left(x,\theta \right)=\frac{1}{2}\left[f\left(x,\theta +\frac{\pi }{2}\right),f\left(x,\theta -\frac{\pi }{2}\right)\right].$$

**Figure 8:**
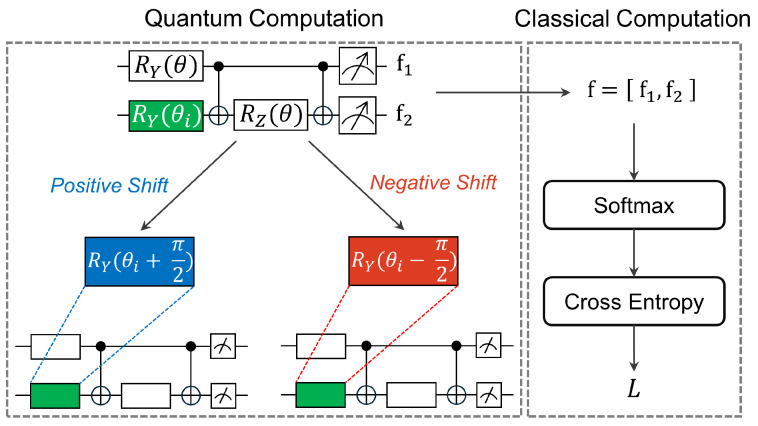
Efficient gradient computation through the technique parameter-shift rules on a VQC.

Figure 8 depicts the procedural steps involved in the technique, i.e., the parameter-shift rules. In each iteration, we perform dual shifts of parameter $\theta_{i}$ by $+\frac{\pi }{2}$ and $-\frac{\pi }{2}$, namely *positive* and *negative* shifts, respectively, and record the measurement results. Subsequently, we apply classical softmax and cross-entropy functions on classical computers to obtain the training loss function *L*.

## 5. Experiments and Results

This section outlines the experimental setup, including details of the dataset, evaluation metrics, experimental environments, and hyperparameters for model adjustment. Subsequently, the experimental results are discussed, focusing on the accuracy and loss functions as the primary metrics. Finally, the hyperparameters of the QLSTM model are adjusted to optimize configuration.

### 5.1. Experimental Settings

We present our experimental setup as a dataset, evaluation metrics, and model hyperparameter.

#### 5.1.1. Dataset

**Figure 9:**
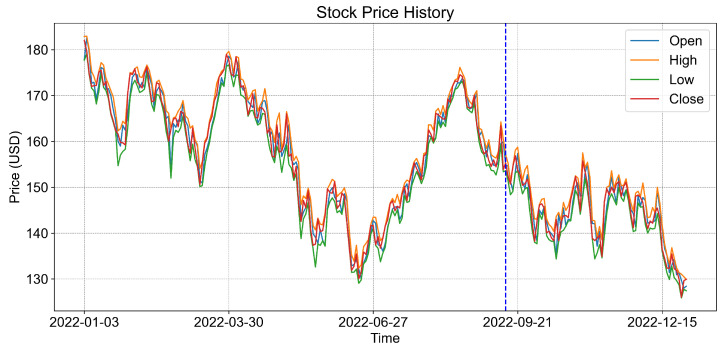
Selected stock price data from 1 January 2022, to 1 January 2023. The training data are depicted on the left side of the blue dashed line, whereas the testing data are on the right side.

We extracted stock price data from Apple Inc. for the period spanning 1 January 2022 to 1 January 2023. The dataset consists of 251 observations collected on weekdays and five columns: Date, Open, High, Low, and Close. To enhance the quality of our analysis, we proceed with data preprocessing for numerical stability and faster convergence, ensuring optimal scaling within the range of [−1, 1]. After preprocessing, we divided the dataset, allocating 70% for training and 30% for testing. Figure 9 shows the stock prices for the chosen period and the corresponding data split.

#### 5.1.2. Evaluation Metrics

We utilized the RMSE and prediction accuracy metrics to evaluate the models. The RMSE quantifies the average magnitude of errors between the predicted values and the actual values, assigning more weight to large errors. The formulas are as follows:

(9) $$\begin{array}{cc} RMSE & =\sqrt{\frac{1}{N}\sum_{t=1}^{N}{\left(y_{t}-\hat{y_{t}}\right)}^{2}}\\ Accuracy & =\frac{1}{N}\sum_{t=1}^{N}y_{t}-\hat{y_{t}} \end{array}$$

where $y_{t}$ is the normalized stock price actual value and $\hat{y}$ is the normalized predicted value for $t^{th}$ data.

#### 5.1.3. Model Hyperparameter

To ensure a rigorous comparison, we designed a classical LSTM to have a number of parameters similar to that of the QLSTM. The LSTM architecture utilizes a hidden size of 7 and contains a single linear layer to convert the output to a predicted value $y_{t}$. The classical LSTM model comprises 288 parameters. For QLSTM, there are 6 VQCs as shown in Figure 5. In each of these VQCs, we utilize 7 qubits, maintaining a depth of 2, with 3 rotations within the variational layer, and an additional 2 parameters for final scaling. Therefore, the number of parameters in QLSTM is 6×7×2×3+2=254. Both the LSTM and QLSTM models are trained using a learning rate of 0.01, the mean squared error (MSE) loss function, and the Adam optimizer across 50 epochs. For other specialized time-series models, we experiment with classical models such as UnSupervised Anomaly Detection for multivariate time series (USAD) [46], Deep Autoencoding Gaussian Mixture Model (DAGMM) [47], Multi-Scale Convolutional Recurrent Encoder-Decoder (MSCRED) [48], and Multivariate Time-series Anomaly Detection via Graph Attention Network (MTAD_GAT) [49]. These models are mainly employed for outlier detection in time series data. However, they are highly dependent on the prediction performance, which makes them strong candidates for comparison. Furthermore, all these models use the same hyperparameters as LSTM and QLSTM.

The proposed QLSTM is implemented using PyTorch and PennyLane [50]. The QLSTM model is trained and simulated using the noiseless IBM simulator. To enhance its fidelity to actual quantum device behaviors, we also train it using a simulator that integrates noise from actual quantum devices into the noiseless environment, known as the noisy IBM simulator. During training, the QLSTM model is solely simulated on the IBM simulator, due to the extended wait times for access to the actual IBM quantum computer. That should be noted that the process of accessing IBM Quantum was achieved through the IBM Quantum platform, which provides cloud-based access to quantum devices. Additionally, for prediction tasks, we utilize an actual IBM quantum computing device, specifically the *IBM Nazca* device. To avoid any confusion, we denote the noiseless IBM simulator as *Noiseless*, the noisy IBM simulator as *Noisy*, and the actual IBM quantum device as *Actual*.

### 5.2. Experimental Results

Here, we present experimental results on the training losses and accuracy of LSTM and QLSTM models, along with other classical models. Thereafter, we compare their stock price prediction performances. Additionally, we explore the performance of QLSTM in various quantum environment scenarios.

#### 5.2.1. Accuracy and Loss

**Figure 10:**
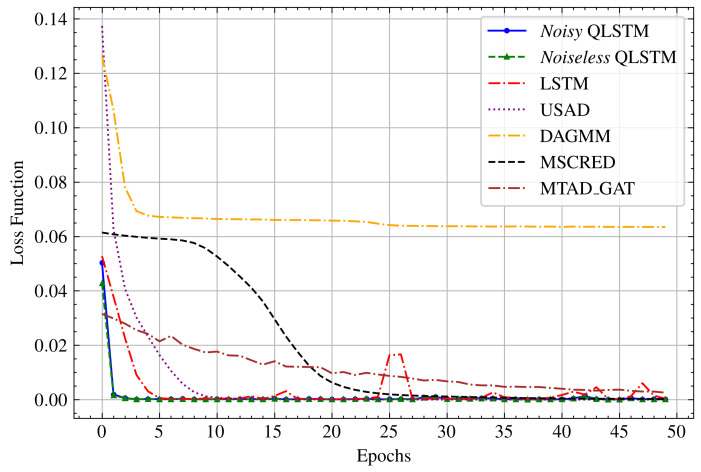
The training losses of the *Noiseless* and *Noisy* QLSTM, classical LSTM, and other models on the stock price dataset over 50 epochs, where the loss function descends to the lowest point near zero.

We commence by exploring the enhanced capabilities of our proposed QLSTM in elevating accuracy and mitigating the training loss. To substantiate the superiority of our QLSTM over traditional LSTM and other models, we conduct a comprehensive comparative analysis of their performance. Figure 10 illustrates the training losses (MSE), indicating that the training losses of the QLSTM consistently remain lower and show less fluctuation than those of LSTM and other models across all epochs. This improvement is attributed to the quantum encoding and representation of classical data within a higher-dimensional Hilbert space, thereby enhancing the data-representation efficiency [51]. In this experiment, QLSTM models are trained in both noiseless and noisy IBM simulator environments over 50 epochs. Despite being trained in the noisy IBM simulator, the *Noisy QLSTM* shows a slightly larger loss function compared to the *Noiseless* variant, which is expected. However, it still performs better than LSTM and other models, respectively. By comparing it with classical LSTM and other models, we can intuitively understand the performance of the proposed QLSTM model and explore its potential for enhancing the handling of long-term dependencies and capturing intricate patterns within sequential data.

Table 1 presents a comparative analysis of accuracy and RMSE loss for both training and prediction across all models. Remarkably, the *Noiseless* QLSTM model outperformed others, achieving a remarkable accuracy score of 1 and an impressively low RMSE loss of 0.0371. Even in the presence of noise, the *Noisy* model surpassed the performance of other classical models with an accuracy of 0.9714 and an RMSE loss of 0.0511, indicating its robustness. However, due to prolonged queuing times for accessing the actual IBM quantum machine [52], we solely performed predictions using the actual IBM quantum machine, thus explaining the unavailability of training accuracy and RMSE. Overall, QLSTM showcased a remarkable approximate 10% enhancement in accuracy, coupled with an impressive 50% reduction in average RMSE compared to classical models.

#### 5.2.2. Prediction Performance

**Figure 11:**
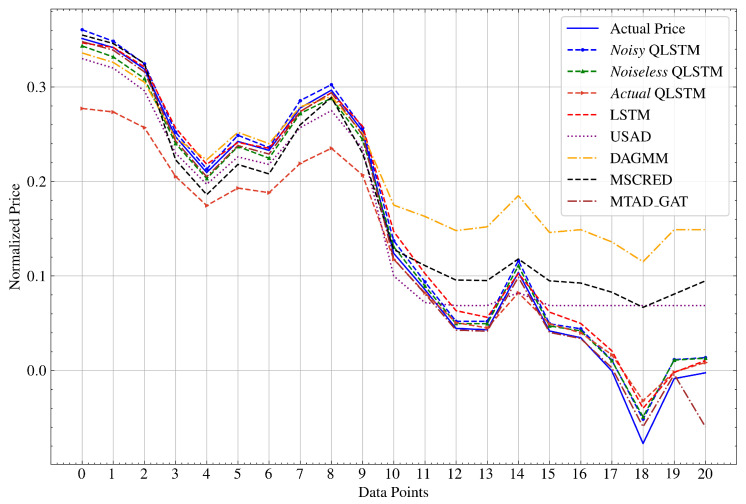
Comparison of the prediction performance of QLSTM in various quantum environments with classical LSTM and other models using 20 stock price data points.

The QLSTM model showcased superior predictive accuracy compared to classical LSTM and other models, with values closely matching the actual stock price data. Figure 11 depicts the comparison of prediction performance. We conducted the comparison using only 20 data points to improve the clarity of the graph. The results highlight a significant advantage of QLSTM in both the Noiseless and Noisy scenarios, consistently outperforming the classical models. However, when predicting within the Actual environment, it has the lowest performance. This observation strongly suggests that the existence of noise in actual quantum machines significantly influences the overall quality of solutions. Besides the discussed loss of accuracy stemming from a limited number of qubits, quantum circuits also encounter quantum noise in practice. This noise arises from quantum processors being susceptible to their environment, leading to quantum decoherence and the loss of their quantum state [53]. Addressing the noise issues requires the implementation of several error-mitigation techniques [54,55], a task that surpasses the boundaries of this study’s scope. One should take note that noisy simulators often outperform actual quantum machines, despite utilizing noise data from actual quantum hardware. This is because simulators typically use a simplified model of the noise that occurs in an actual quantum machine. Real-world noise can be complex and come from various sources. Simulators may not capture all the intricacies of this noise, leading to slight differences in behavior [56]. The rapid convergence underscores the QLSTM model’s exceptional ability to adapt swiftly to the inherent patterns within the data, a characteristic that sharply distinguishes it from traditional LSTM and other models. This model, with its highly accurate and reliable predictions, holds the potential to revolutionize stock price predictions in financial applications.

#### 5.2.3. Number of Qubits

**Figure 12:**
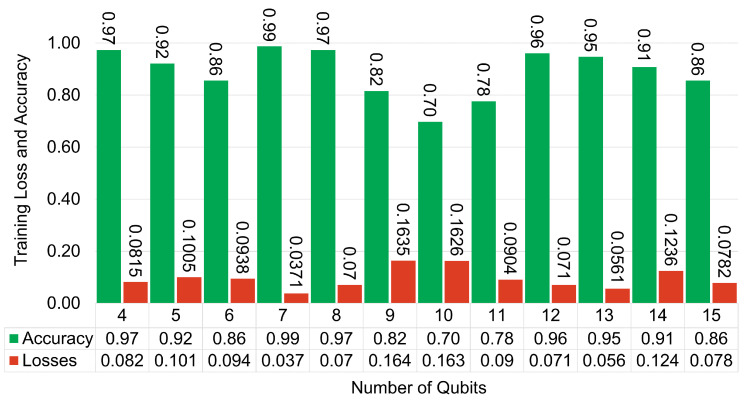
Visualizing the training accuracy and loss of QLSTM models across different numbers of qubits, spanning from 4 to 15. Green bars represent accuracy, while red bars denote losses in RMSE.

We conduct a study on the impact of the number of qubits on the prediction performance of Noiseless QLSTM. Figure 12 illustrates that despite the increasing number of qubits, the corresponding results do not show significant improvement. In some cases, such as the transition from 8 to 11 qubits, the performance appears to degrade, even as the complexity of the quantum circuits increases. One plausible explanation for the lower results could be attributed to a phenomenon known as the barren plateau problem during training. The barren plateau phenomenon is a well-known challenge encountered in VQCs during the optimization of parameters using classical optimization algorithms [57]. This phenomenon occurs when the optimization landscape becomes exceedingly complex, leading to exponentially vanished gradients as the number of qubits within the circuit increases [58]. This finding indicates that the choice of the number of qubits should be carefully chosen, aligning with the complexity of the problem and the requirements of the quantum algorithm.

## 6. Discussion

The results of contrasting QLSTM with other models and the standard LSTM highlight the advantages of QML in increasing prediction accuracy and decreasing the loss function in stock-price analysis. However, further investigations are necessary to validate these findings conclusively. One significant advantage of the QLSTM model is its utilization of quantum data encoding, which translates classical data into quantum states and represents them within superposition and higher-dimensional spaces known as the Hilbert space. This space is leveraged for the efficient manipulation and processing of complex information, enabling quantum algorithms to tackle computational tasks that would otherwise be intractable for classical computers. This facilitates advancements in fields, such as optimization and ML tasks. Notably, although significant model improvements were achieved, our study has several limitations.

1. Dataset Scope: Given the limitations of quantum simulations on classical computers and the prolonged queuing time for accessing the IBM actual quantum machines, we were restricted to utilizing only 251 stock-price data points. Even though the proposed model delivered promising performance, further experimentation using larger datasets is warranted.
2. Model Design: Our model architecture is configured with specific hyperparameters, a specific quantum data-encoding layer, quantum rotation gates, and a particular type of variational layer to refine the prediction task. However, extensive investigation is required for in-depth understanding, including diverse quantum circuit designs, variations in gate types and quantities, and exploration into the depth of the variational layer.
3. Simulation Limitations: We initially employed a few qubits. However, given the current availability of quantum devices with hundreds of qubits, it is recommended that we consider evaluating the QLSTM model with large datasets and qubit numbers to provide a more comprehensive assessment of its real-world performance.
4. Possibility of Classical Simulation: While variational quantum circuits are promising for quantum advantage, recent studies suggest that certain types of VQCs, both noisy and noiseless, can be simulated on classical computers in polynomial time. For small to medium problem sizes, classical simulation may achieve comparable results without the overhead of quantum hardware. However, as we move towards larger, more complex tasks, the polynomial scaling of quantum models is expected to surpass classical capabilities, particularly when combined with specialized quantum hardware. In this study, we focus on cases where quantum circuits maintain an edge in efficiency and predictive accuracy. Nevertheless, we acknowledge the importance of identifying the boundaries where quantum computation significantly outperforms classical simulation to fully validate the advantage of our approach [59,60].

In summary, beyond the domain of stock-price prediction, the capabilities of QLSTMs suggest that they can be applied in other sectors, such as renewable energy and the Internet of Things, as they generally handle time-series data. Resolving the outlined limitations could highlight the advantages of QLSTMs in real-world prediction applications.

## 7. Conclusions

We present a hybrid quantum-classical computing framework that leverages the classical LSTM model for highly accurate stock price prediction. The QLSTM integrates the classical LSTM architecture with VQCs to enhance model learning by substituting LSTM gates with quantum circuits, specifically VQCs. To validate the model’s performance, we conducted experiments using an IBM simulator running on a classical computer, a noisy IBM simulator embedded with quantum noise from an actual quantum machine, and the actual IBM quantum machine. In our experiments, we compared the performance of classical LSTM and QLSTM in terms of training and prediction losses, accuracy, and prediction performance. Specifically, we investigated the impact of the number of qubits on the performance of the QLSTM model. The results showed that QLSTM outperforms classical LSTM and other models with significantly lower RMSE and higher accuracy in both training and prediction tasks. Overall, the QLSTM model achieved a 50% reduction in RMSE and a 10% improvement in accuracy compared to LSTM. The results also show that with fewer parameters, QLSTM outperformed classical LSTM models with many parameters. However, regarding evaluations on an actual quantum machine, the QLSTM model performed worse compared to its classical counterparts. This study is among the first to successfully predict stock prices using a hybrid quantum-classical computing framework. Nevertheless, while recognizing that quantum circuits are yet to be fully operational for inference and practical applications, this study lays the foundation for implementing NNs within a QC framework. Notably, currently, state-of-the-art and out-of-the-box classical ML techniques remain formidable and steadily outperform QC techniques. Future work will focus on expanding the QLSTM model’s scalability by exploring more advanced quantum error mitigation techniques to address the limitations posed by noise in actual quantum machines. Additionally, we aim to experiment with deeper variational quantum circuits to assess their impact on model expressiveness and generalization, while carefully managing the risk of barren plateaus. Another promising direction is the integration of quantum-inspired algorithms that can bridge the gap between quantum and classical models, potentially enabling quantum-like performance on classical hardware for smaller-scale applications. Finally, we plan to extend the application of QLSTM beyond stock price prediction to other time-series domains, such as energy forecasting and healthcare, to further validate its robustness and versatility in diverse predictive tasks.

## Figures and Tables

**Table 1:** Comparison of the training and prediction accuracies, as well as the RMSE loss values, of the QLSTM model in different quantum environments with those of the classical LSTM and other models. N/A denotes “not available”.

Models	Training Acc	Training RMSE	Prediction Acc	Prediction RMSE
*Noiseless* QLSTM	**1.00**	**0.0371**	**0.9736**	**0.0602**
*Noisy* QLSTM	0.9714	0.0511	0.9210	0.0648
*Actual* QLSTM	N/A	N/A	0.7619	0.1401
LSTM	0.92	0.0567	0.8815	0.0693
QSVM [36]	N/A	N/A	0.5894	N/A
USAD [46]	0.9342	0.0708	0.8874	0.0672
DAGMM [47]	0.8947	0.0768	0.8410	0.0721
MSCRED [48]	0.9342	0.0720	0.8828	0.0680
MTAD_GAT [49]	0.9473	0.0668	0.8857	0.0624

## Data Availability

The source code used in this study, implemented using PyTorch and PennyLane, is available at the GitHub repository https://github.com/QCL-PKNU/SPP-QLSTM (accessed on 18 August 2024).
